# Case Report: Clinical pharmacist prescriber in depression treatment in primary care settings: clinical case focused on prescribing practice

**DOI:** 10.3389/fpsyt.2025.1677152

**Published:** 2025-08-28

**Authors:** Matej Stuhec

**Affiliations:** ^1^ Department of Pharmacology & Department of Clinical Pharmacy, Medical Faculty Maribor, University of Maribor, Maribor, Slovenia; ^2^ Department of Clinical Pharmacy, Ormoz’s Psychiatric Hospital, Ormoz, Slovenia

**Keywords:** depression, pharmacist prescriber, clinical pharmacy, ambulatory care, medication review

## Abstract

**Introduction:**

Pharmacotherapy of depression represents a significant challenge in the management of depression in primary care. Although effective treatments have been available, many patients are still not adequately managed. Clinical pharmacists represent one of the possible strategies in the management, although this practice is rarely seen outside the United Kingdom and the United States.

**Aim:**

The aim of the case was to evaluate the impact of clinical pharmacist prescribers on depression treatment.

**Methods:**

A longitudinal, observational, case-based medication review by a pharmacist prescriber was conducted for a 63-year-old Slovenian patient in a primary care ambulatory setting. The review included three structured medication review assessments performed by a clinical pharmacist prescriber at defined intervals: first observation, two months post-intervention, and six months after first observation. The pharmacists conducted medication reviews and prescribed medications like physicians, operating within a collaborative practice agreement as dependent prescribers. Predefined outcomes included diabetes management (HbA1c and blood glucose), lipid levels (S-LDL), pain (Visual Analogue Scale [VAS]), depression (Patient Health Questionnaire-9 [PHQ-9]), and quality of life (assessed via EQ-5D-VAS). The patient’s complete medication regimens were reviewed, focusing on dosage appropriateness, indication matching, potential drug-drug interactions, and medication adherence.

**Results:**

A 63-year-old male Slovenian patient diagnosed with depression, type 2 diabetes with polyneuropathy, and hypothyroidism underwent two medication reviews between December 2024 and July 2025. The pharmacist prescribed amitriptyline and semaglutide (accepted by the patient’s physician). Notable improvements were observed in glycemic control (HbA1c reduced from 9.9% to 8.2%), and quality of life (EQ-5D-VAS score improved from 30/100 to 80/100). Depression symptoms also resolved, with the PHQ-9 score improving from 11 to 4.

**Conclusions:**

This case study demonstrates that interventions by a clinical pharmacist prescriber during the medication review process resulted in improved clinical outcomes in the treatment of depression, as well as enhanced quality of life. It represents an important contribution to the development of pharmacist prescribing roles in depression management within primary care settings outside of the United Kingdom and the United States.

## 1 Introduction

Major depressive disorder (depression) represents a significant global disease burden (1). According to the World Health Organization (WHO) report, depression is especially prevalent in primary care, where its burden is increasing significantly. WHO projections indicate that by 2030, depression will be the leading cause of disease burden worldwide (2). Depression rates in Central Europe, including Germany, have been rising. In Germany, the 12-month prevalence of unipolar depression is estimated at approximately 7.7%, with major depression accounting for around 6.0% of the population (3).

Although effective pharmacological and non-pharmacological treatment options are available, many patients still do not receive adequate care (4). According to a study by Kessler and colleagues, fewer than 50% of patients with depression in primary care receive adequate treatment (4). The study reported that 51.6% of patients with 12-month depression received healthcare treatment, and of these, only 41.9% received adequate care (4). Similar challenges exist in Germany, where most general practitioners (GPs) report poor communication with psychiatrists. GPs are responsible for diagnosing and managing the majority of depression cases in primary care, with 64.1% of outpatient incidental depression patients receiving treatment exclusively from GPs. A significant barrier to effective depression management in primary care is the lack of collaboration between GPs and psychiatrists (5).

Furthermore, adherence to depression treatment guidelines is often poor. For example, in primary care in the Netherlands, adherence to treatment guidelines was only 42% (6). German researchers have emphasized the urgent need for collaborative healthcare models to address obstacles arising from fragmented mental health care systems. In cases of inappropriate treatment or progression of depression, patients are at risk of developing treatment-resistant depression (TRD), which is significantly more challenging to manage. TRD often requires augmentation strategies, such as the addition of lithium, antipsychotics, or esketamine (7, 8).

Furthermore, depression is two to three times more prevalent among individuals with multimorbidity. The presence of multiple chronic conditions complicates depression management and often limits adherence to clinical guidelines, which are typically not designed with this patient population in mind. A systematic review included 40 studies that found a weak but statistically significant association between the number of chronic conditions and the severity of depressive symptoms [r = 0.26 (95% CI 0.18–0.33), p < 0.001] (9).

These limitations and existing gaps in the treatment of depression, particularly in patients with multimorbidity, highlight the pressing need for more effective, interdisciplinary collaboration in primary care. Involving a broader range of healthcare specialists, including clinical pharmacist prescribers, may provide a valuable strategy to enhance treatment outcomes and address current deficiencies in care delivery. Traditionally, psychiatrists have treated depression, but its high prevalence has shifted much of the treatment responsibility to the primary care level (2, 4). In some countries, including Slovenia and Germany, most antidepressants and other related medications are prescribed by general practitioners (GPs) (5, 10). In this context, primary care represents a crucial setting for collaboration between GPs and clinical pharmacists in treating depression (10).

Clinical pharmacists collaborate with GPs in various ways, including conducting medication reviews and, in some cases, prescribing (11, 12). The authors of the position paper highlighted that clinical pharmacists are not adequately integrated into mental health care, including the treatment of depression, and proposed the establishment of nationally reimbursed services to address this gap. In several European countries, the role of clinical pharmacists in depression management remains underrecognized, and they are often not included as members of multidisciplinary care teams. In this context, the authors emphasized the importance of implementing reimbursed clinical pharmacy services, citing Slovenia as an example where clinical pharmacists provide medication reviews at the national level. Additionally, they referred to the United Kingdom, where pharmacist prescribers are an established part of the primary care system (12).

Numerous trials have demonstrated that clinical pharmacists can improve adherence to treatment guidelines through medication reviews, even when they do not have prescribing authority (10). Medication reviews by clinical pharmacists are among the most effective strategies to optimize depression treatment. For instance, a study by Stuhec and Lah showed that interventions through medication reviews in Slovenian ambulatory settings in a primary health center led to a 40% increase in adherence to depression treatment guidelines—a significant improvement (n=30 patients) (13). The acceptance rate of GPs was 55%, and most of the recommendations were based on medication switching and dose adjustments (13). These studies demonstrate that clinical pharmacists’ medication reviews in ambulatory settings within primary health centers contribute to improved treatment outcomes and may support more effective management of depression. Pharmacist prescribers represent an important additional resource, potentially enhancing care through further prescription, either independently (without prior approval) or dependently (in collaboration with a physician’s permission).

Although prescribing has traditionally been the domain of physicians, this role has expanded to include other healthcare professionals such as clinical pharmacists and nurses (14). Clinical pharmacists have been recognized as independent prescribers in the United Kingdom for over 20 years (15). In the United States, clinical pharmacists prescribe medications through various protocols (e.g., Collaborative Care Agreements), allowing them to prescribe for depression in some regions (16). Pharmacist prescriber roles are also being developed in Australia, New Zealand, and Slovenia (10, 17).

The European Society of Clinical Pharmacy (ESCP), in its position paper, emphasized the need for clinical pharmacists to develop the competencies required for prescribing in mental health, including depression, across Europe and beyond (12). They noted that pharmacist prescribing in mental health remains underdeveloped, except in the UK and certain parts of the US (12).

In this context, the main aim of this paper is to present a case of a patient with depression in which a pharmacist prescriber provided medication review, additional dependent prescribing, and ongoing monitoring. A secondary aim is to present the rationale for the global development of such services. We acknowledge that this case description does not constitute a full study but serves as an important starting point for the development of the pharmacist prescriber role.

## 2 Methods

A longitudinal, observational, case-based medication review by a clinical pharmacist was conducted for a 63-year-old Slovenian patient in a primary care ambulatory setting. Patients were referred to the pharmacist prescriber by GPs based on clinical complexity, such as the presence of depressive symptoms and multimorbidity, as well as medication-related problems, including critical drug-drug interactions and polypharmacy. The review included three structured medication review assessments performed by a clinical pharmacist at defined intervals: first observation, two months post-intervention, and six months after first observation. The pharmacists conducted medication reviews and prescribed medications like physicians, operating within a collaborative practice agreement as dependent prescribers. Predefined outcomes included diabetes management (HbA1c and blood glucose), lipid levels (S-LDL), pain (Visual Analogue Scale [VAS]), depression (Patient Health Questionnaire-9 [PHQ-9]), and quality of life (assessed via EQ-5D-VAS). The patient’s complete medication regimens were reviewed, focusing on dosage appropriateness, indication matching, potential drug-drug interactions, and medication adherence.

The patient is part of a national pre-post prospective study involving clinical pharmacist prescribers working in primary care ambulatory settings in Slovenia. The clinical pharmacist prescriber conducts a medication review (advanced medication review, type 3 according to the Pharmaceutical Care Network Europe-PCNE) and may initiate or adjust therapy as needed (extra service to medication review) (10, 11). Medication reviews type 3 (advanced medication review) have been reimbursed in Slovenia and recognized as a pharmacist service since 2017, but clinical pharmacists do not have prescribing rights (10). Medication review type 3 is based on a patient’s medication history, relevant patient information, and clinical data. It addresses all critical aspects outlined by the PCNE, including drug interactions, side effects, unusual dosages, adherence issues, drug-food interactions, effectiveness concerns, over-the-counter medication problems, unindicated medications, missing indications, and dosage issues (10, 11). This study researched clinical pharmacists prescribers. In this study, clinical pharmacists can prescribe medications under a collaborative agreement, which must be approved by both the GP and the patient before prescribing and monitoring begin. Consent for participation in the study may also be cancelled by the patient or the physician for the duration of the study.

Clinical pharmacist prescribers may prescribe and monitor medications listed in the collaborative practice agreement until the third patient visit (six months after the initial visit). After each prescription by the clinical pharmacist prescriber, the GP must approve the prescription, making this a pharmacist-dependent prescribing model. GPs make a final decision on all prescription acceptance. In 2024, the Slovenian National Medical Ethics Committee granted ethical approval for the study (N#0120-330/2024-2711-3). Informed consent was obtained from this patient. The CARE guidelines were followed in the preparation of this manuscript.

## 3 Case report

A 63-year-old Slovenian male patient with a diagnosis of major depressive disorder, type 2 diabetes mellitus complicated by peripheral polyneuropathy, obesity (body weight >120 kg), and hypothyroidism underwent three structured medication reviews on 4 December 2024, 11 February 2025, and 3 July 2025. His medical history included major depressive disorder, angina pectoris, hypertension, insomnia, neuropathic pain, and type 2 diabetes. Laboratory results collected during the first pharmacist visit showed a normal complete blood count, normal liver enzymes, and normal liver function tests. However, the serum creatinine level was elevated, and the glomerular filtration rate (GFR) was calculated at 46 mL/min. The patient also had elevated levels of glycated hemoglobin (HbA1c, 9.9%) and triglycerides (4.97 mmol/L; normal 0.6-1.7 mmol/L). There was no history of dementia or smoking.

Patient was treated with multiple medications, including pregabalin 300 mg daily, quetiapine 25 mg at bedtime, vortioxetine 15 mg daily, furosemide 40 mg daily, pantoprazole 20 mg daily, levothyroxine 25 mcg daily, aspirin 100 mg daily, perindopril/indapamide 8 mg/2.5 mg daily, rosuvastatin/ezetimibe 20 mg/10 mg daily, dapagliflozin/metformin 5 mg/1000 mg twice daily, two types of insulin (as part and glargine), trimetazidine 35 mg twice daily, and fenofibrate 250 mg daily. The GP was not fully satisfied with the clinical outcomes (e.g., depression, elevated HbA1c and polyneuropathy) and referred the patient to the clinical pharmacist in December 2024 for medication review. In addition, the GP specified in the collaborative practice agreement that clinical pharmacists could prescribe, modify or discontinue all medications within the medication list, including medication initiation if necessary and monitor patients for up to 6 months.

At the initial visit (4 December 2024), clinical pharmacists conducted a comprehensive medication review and initiated changes to pharmacotherapy. Modifications were prescribed due to suboptimal therapeutic outcomes in the management of depression, diabetes, and pain. Depression symptoms were assessed using the PHQ-9, with a score of 11 indicating the absence of remission. Health-related quality of life was evaluated using the EQ-5D Visual Analogue Scale (VAS), with a score of 80/100, and pain was assessed using the VAS, with the patient reporting severe pain intensity (VAS score: 10/10).

Based on the assessment, the clinical pharmacist initiated amitriptyline at 25 mg twice daily with a plan to titrate to 50 mg twice daily and semaglutide at 3 mg daily, increasing to 7 mg daily after two weeks. Quetiapine was discontinued. The patient’s GP accepted all proposed medication changes.

At the follow-up visit on 5 February 2025 (two months after the initial consultation), the clinical pharmacist reassessed treatment outcomes and conducted a second medication review. The patient reported marked improvements, particularly in depressive symptoms and pain. Objective improvements included a reduction in HbA1c from 9.9% to 8.2%, an increase in estimated GFR from 46 to 63 mL/min, improved quality of life (EQ-5D-VAS score 80/100), and decreased pain intensity (VAS score: 5/10). The PHQ-9 score decreased from 11 to 4, indicating remission of depressive symptoms. No additional pharmacological changes were recommended at this visit. However, the clinical pharmacist provided counselling on the importance of adherence to fenofibrate therapy, as the patient reported inconsistent use, which was reflected in elevated triglyceride levels (9.5 mmol/L).

A third medication review was conducted at the third visit on 5 July 2025 (six months after the initial consultation). The patient reported sustained improvement compared to the baseline visit, with outcomes consistent with those observed at the second visit. Glycemic control improved (HbA1c: 8.8% vs 9.9% at baseline), and depressive symptoms remained in remission with a PHQ-9 score of 4. Pain intensity further decreased (VAS score: 3/10). The EQ-5D-VAS score was 80/100 at the third visit. Triglyceride levels improved significantly, decreasing to 2.8 mmol/L. No further pharmacotherapy adjustments were deemed necessary. A summary of the case report, including key outcomes, is presented in the **Table 1**.

**Table 1:** Summary of the case report: key dates, medications, and clinical outcomes.

Outcome scales	Medication review N#1	Medication review N#2	Medication review N#3
Date	4 December 2024	4 December 2024	4 December 2024
Medication Changes	Pharmacist initiated amitriptyline at 25 mg twice daily with a plan to titrate to 50 mg twice daily and semaglutide at 3 mg daily, increasing to 7 mg daily after two weeks. Quetiapine was discontinued. Accepted by the general practitioner.	No additional pharmacological changes were recommended at this visit. However, the clinical pharmacist provided counselling on the importance of adherence to fenofibrate therapy.	No changes. No additional pharmacological changes were recommended.
Levels of glycated hemoglobin (HbA1c)	9.9%	8.2%	8.8%
Patient Health Questionnaire-9 (PHQ-9) score	11/27 (no remission)	4/27 (remission)	4/27 (remission)
EQ-5D Visual Analogue Scale (VAS) score	10/10	5/10	3/10
EQ-5D visual analogue scale (EQ-5D-VAS) score	30/100	80/100	80/100
Triglyceride levels	4.97 mmol/L	9.5 mmol/L	2.8 mmol/L

## 4 Discussion

This case report highlights the potential for clinical pharmacist prescribers to contribute to improved clinical outcomes, which constitutes the primary objective in the management of depression. In Slovenia, clinical pharmacists have been integrated into the healthcare system since 2017, where their role primarily focus on medication review without prescribing (10). In this context, the present case introduces a novel approach compared to previous Slovenian studies (10), where clinical pharmacists were limited to conducting medication reviews, and the role of pharmacist prescribers had not yet been described. The integration of pharmacist prescribers represents a significant advancement in collaborative care within primary care settings. Future studies will involve a larger number of patients managed by pharmacist-dependent prescribers, which will also open the way for investigating the role of pharmacist-independent prescribers in clinical practice.

Patients with depression frequently present with multimorbidity, and this complex case illustrates how clinical pharmacists—through medication review, ongoing monitoring, and prescribing—can support GPs in achieving favorable clinical outcomes. In this case, both depression and type 2 diabetes with associated polyneuropathy improved, with remission achieved. In addition, the patient and the GP reported that quality of life improved significantly by approximately 50%. The positive impact of clinical pharmacist interventions on quality of life was also demonstrated in our previous study involving 24 patients, in which clinical pharmacists monitored patients without having prescribing authority (18). The case also demonstrates significant clinical improvements, as the patient’s quality of life increased by 50% on the EQ-5D-VAS, which exceeds the threshold for clinical significance (19). This improvement was further supported by clinical remission on the PHQ-9 and was corroborated by the patient’s self-reported improvements.

The prevalence of pain in patients with depression is estimated to be approximately 65%, according to a pooled analysis of multiple studies (20). This highlights the complexity often encountered in primary care and underscores the potential role of clinical pharmacists in optimizing pharmacotherapy. In this case, the clinical pharmacist prescribed amitriptyline, an antidepressant, following clinical guidelines for pain and depression treatment (21). Additionally, semaglutide was prescribed to support glycemic control and weight management, particularly relevant for this patient with obesity (weight >120 kg) and type 2 diabetes. The intervention significantly reduced HbA1c levels, consistent with evidence-based recommendations for using GLP-1 receptor agonists in this patient population (22). The pharmacist prescriber also educated the patient on medication adherence, which contributed to a significant decrease in the patient’s triglyceride levels by the final visit. The patient had not taken fenofibrate between the first and second visits, which explained the elevated triglyceride levels observed at that time.

Evidence from primary care settings suggests that medication reviews conducted by clinical pharmacists in the context of mental health care are associated with favorable outcomes, including reductions in polypharmacy, fewer drug-drug interactions, and enhanced adherence to treatment guidelines (13, 14). In addition to conducting medication reviews, clinical pharmacists are authorized to prescribe guideline-recommended pharmacotherapy and to monitor patients longitudinally in some countries. This model of care remains novel in many European countries, where medication prescribing and monitoring have traditionally been the sole responsibility of physicians. Notably, the United Kingdom has been recognized for expanding the scope of clinical pharmacists, including prescribing for depression (23). This case highlights that pilot trials involving pharmacist prescribers are also feasible and valuable in countries where clinical pharmacists do not yet have prescribing rights. In Slovenia, where this service is currently in development, a pilot trial has been approved and conducted. In contrast, clinical pharmacy in other Central European countries has not reached the same level of advancement as in Slovenia (24). In Slovenia, three key clinical pharmacy services—delivered in ambulatory primary care, hospital outpatient settings, and through seamless care models—are reimbursed by the national insurance company and well-established, providing a crucial foundation for the development of pharmacist prescribing roles (23). Notably, clinical pharmacy services in Slovenia are more developed than in some wealthier neighboring countries, such as Italy and Austria (24).

A 12-month pilot study conducted in Scotland involving 75 patients demonstrated that clinical pharmacists, acting as independent prescribers, were able to initiate and modify pharmacological treatment for depression and generalized anxiety disorder (GAD) without requiring direct referral to GPs. Pharmacological interventions included antidepressants and anxiolytics. The study reported clinical remission or treatment response in most patients, with reductions in PHQ-9 and GAD-7 scores by 45% and 50%, respectively. Pharmacists prescribed treatment following diagnoses established by GPs (23). In a randomized controlled trial conducted in a primary care setting in the United States, Finley et al. evaluated the outcomes of 75 patients managed by clinical pharmacist prescribers compared with 50 patients receiving standard care. Pharmacists operated as dependent prescribers under a collaborative practice agreement in an ambulatory care environment. After six months, the intervention group demonstrated significantly higher medication adherence rates than the control group (67% vs. 48%; odds ratio = 2.17; 95% CI: 1.04–4.51; P = 0.038). Patient satisfaction scores were significantly greater in the intervention group, and provider satisfaction was also high. Although clinical improvement was observed in both groups, the between-group difference was not statistically significant (25). Another study in the United States evaluated the impact of clinical pharmacists acting as dependent prescribers under collaborative practice agreements. This prospective, nonrandomized proof-of-concept study was conducted from July 2006 to December 2007 and included 151 patients with depression. Statistically significant reductions were observed in PHQ-9 scores from baseline to endpoint (11.5 ± 6.6 to 5.3 ± 4.7; P < 0.0001). The clinical response rate was 68%, with a remission rate of 56%. Moreover, the intervention was associated with a reduction in projected annual healthcare costs per patient (16).

Comparable findings were reported by Adler et al. in a 6-month randomized study involving 533 patients with depression and/or dysthymia in U.S. primary care settings (26). In this trial, clinical pharmacists provided in-person and telephone consultations, supporting GPs and patients in selecting, dosing, and adjusting antidepressant therapy. Antidepressant utilization rates at six months were significantly higher in the intervention group than in controls (57.5% vs. 46.2%; P = 0.03). However, differences in symptom severity did not reach statistical significance (26).

In addition to the studies previously mentioned, a meta-analysis examining the impact of pharmacists on depression treatment has been published, including 12 studies and a total of 2,133 patients (18). The results demonstrated a significantly higher number of patients with good adherence in the pharmacist intervention group compared to usual care (relative risk = 1.39; 95% CI: 1.11–1.75), as well as improved medication adherence scores (standardized mean difference = 0.32; 95% CI: 0.07–0.56). However, no statistically significant differences were observed in clinical rating scales or quality of life measures (27). The meta-analysis did not restrict inclusion to studies where pharmacists were authorized to prescribe medications; instead, it included a wide range of pharmacist-led interventions, including medication therapy management, adherence counselling, and educational support related to depression and antidepressants.

Several limitations of this case should be acknowledged. The findings from a single case cannot be generalized, which represents a significant limitation of this study. The findings are derived from a single case report, and further studies with larger sample sizes are necessary to confirm these results. The follow-up period was limited to six months, as defined by the scope of a pilot trial approved by the Slovenian National Medical Ethics Committee. Additionally, clinical pharmacists in this case did not have independent prescribing authority, as such rights are not currently granted in Slovenia. This limitation may have constrained the potential impact and evaluation of the intervention. This case should be replicated in studies with larger sample sizes to confirm the findings.

Nonetheless, the case highlights several positive aspects. Over the past decade, the role of clinical pharmacists in Slovenia has expanded significantly, with the introduction of reimbursed clinical pharmacy services such as medication reviews and reconciliation. Incorporating prescribing rights would represent a logical step in enhancing medication review services. This case demonstrates that clinical pharmacists, collaborating with GPs, can effectively monitor patients and contribute to improved treatment outcomes. These findings align with the Committee of Ministers’ Resolution CM/Res (2020)3 on the Implementation of pharmaceutical care for the benefit of patients and health services and with the principles endorsed by the European Society of Clinical Pharmacy (28, 29).

This case also highlights that established and reimbursed medication review services within the country provide a necessary starting point for the development of pharmacist prescriber roles. General practitioners are already familiar with clinical pharmacy practices, including medication reviews, and have established effective team-based collaboration with ambulatory clinical pharmacy services in primary care settings.

## 5 Conclusion

This case report demonstrates that an ambulatory clinical pharmacist prescriber can effectively contribute to improved clinical outcomes in the treatment of depression through collaborative care with GPs in primary care settings. Such collaboration has the potential to address existing treatment gaps and enhance patient monitoring in depression management. Although these findings are encouraging, a larger-scale clinical study is necessary to confirm or refute these results.

## Data Availability

Publicly available datasets were analyzed in this study. This data can be found here: The original contributions presented in the study are included in the article/supplementary material. Further inquiries can be directed to the corresponding author.
